# Enhanced Control of Bladder-Associated Tumors Using Shrimp Anti-Lipopolysaccharide Factor (SALF) Antimicrobial Peptide as a Cancer Vaccine Adjuvant in Mice

**DOI:** 10.3390/md13053241

**Published:** 2015-05-21

**Authors:** Han-Ning Huang, Venugopal Rajanbabu, Chieh-Yu Pan, Yi-Lin Chan, Jyh-Yih Chen, Chang-Jer Wu

**Affiliations:** 1 Department of Food Science, National Taiwan Ocean University, Keelung 202, Taiwan; E-Mail: henryhw910@yahoo.com.tw; 2 Marine Research Station, Institute of Cellular and Organismic Biology, Academia Sinica, 23-10 Dahuen Road, Jiaushi, Ilan 262, Taiwan; E-Mail: ratcatflat@yahoo.com; 3 Department and Graduate Institute of Aquaculture, National Kaohsiung Marine University, Kaohsiung 811, Taiwan; E-Mail: panjade@webmail.nkmu.edu.tw; 4 Department of Life Science, Chinese Culture University, Taipei 111, Taiwan; E-Mail: zyl13@ulive.pccu.edu.tw

**Keywords:** SALF, antimicrobial peptide, MBT-2, bladder tumor, cancer vaccine

## Abstract

Shrimp anti-lipopolysaccharide factor (SALF) is an antimicrobial peptide with reported anticancer activities, such as suppression of tumor progression. In this study, we prepared a potential cancer vaccine comprised of SALF in conjunction with the cell lysate of inactivated murine bladder carcinoma cells (MBT-2), and evaluated its efficacy in a mouse tumor model. Our study shows that SALF added to cell culture media inhibits growth progression of MBT-2, and that SALF together with inactivated MBT-2 lysate elevates the level of inflammasome activity, and modulates the levels of IL-1β, MCP-1, IL-6, IL-12, and TNF-α in mouse macrophages. Immunization of 7, 14, and 21 day-old mice with the vaccine prevented growth of MBT-2 cell-mediated tumors. The vaccine was found to enhance expression of T-cell, cytotoxic T cells, and NK cells in the immunized mice groups. Recruitment of macrophages, T-helper cells, and NK cells was enhanced, but levels of VEGF were decreased in immunized mice. This report provides empirical evidence that our SALF as vaccine adjuvant enhances antitumor immunity in mice.

## 1. Introduction

Antimicrobial peptides (AMPs) play an important role as first-line defensins, warding off the invasion of pathogens [1]. One such AMP, shrimp anti-lipopolysaccharide factor (SALF), was identified from the hemocytes of the shrimp *Penaeus monodon* [2,3]. SALF possesses a broad spectrum of anti-microbial activities, effective against filamentous fungi and Gram-positive/-negative bacteria [4,5]. In aquatic species, SALF has strong anti-viral activity against white spot syndrome virus, fish nodavirus, and yellow head virus [6,7,8]. SALF has also been shown to reduce mouse mortality through *Pseudomonas aeruginosa* infection [9]. Furthermore, SALF induces cell apoptosis (through the death receptor in tumor cells), activates caspases-6, -7, and -9, and down regulates bcl-2 and nuclear factor (NF)-κB [10]. In addition to its *in vitro* anti-tumorigenic activities, SALF has an intrinsic ability to diminish tumor xenografts in mouse models.

Bladder cancer accounts for about 2% of cancer-associated mortality, and is the fifth most common cancer globally [11]. Uncontrolled proliferation of cancer cells and tissue invasion by angiogenesis cause tumor formation [12]. In addition to radiotherapy and surgery, chemotherapy is a primary strategy for treating cancers [13]. However, development of drug resistance, biotransformation, improper biodistribution, poor drug clearance, and inability to target drug delivery to tumor cells are the key challenges faced by chemotherapy [14]. Overcoming such obstacles is the subject of extensive research, from which immune therapy directly targeted to cancer cells has emerged as a promising treatment [15].

Tumor-associated antigens (TAAs) may be the key to developing both humoral and cell-mediated immune therapies against cancers [16]. The first step towards developing tumor immunity involves the migration of monocytes, macrophages, or dendrite cells. Secretion of the chemokine MCP-1 generally attracts monocytes or macrophages (cell-mediated immunity) [17], while the proinflammatory cytokine IL-6 activates B cells to produce tumor-specific antibodies (humoral immunity) [18]. Additionally, cytokine IL-12 may up-regulate IFN-γ in NK and T cells, which promote T-cell differentiation towards Th1-type immunity [19], while IL-10 may down-regulate IFN-γ [20]. Tumor-associated macrophages (TAM) derived from peripheral blood monocytes may be recruited into the tumor tissues [21]. Upon activation by cancer antigens, TAM are able to release a host of immune effectors, including growth factors, proteolytic enzymes, cytokines, and inflammatory mediators [22]. On the other hand, neutrophils promote tumor progression via matrix degradation, immunosculpting, tumor cell proliferation, increased metastasis, and enhanced angiogenesis [23]. Ablation of IL-10 increases tumor incidence, growth, and metastasis [24]. Cancer vaccine therapy is a contemporary therapeutic development, which focuses on eliciting cytotoxic T cells (CTLs) [25]. T-helper cells activated by IFN-γ in conjunction with antigen-presenting cells (APC) enhance antigen presentation to CTLs [26]. Importantly, activated APC may present tumor antigens in lymph nodes to promote tumor specific CTLs [27]. NK cells are potent effectors that express a set of activating and inhibitory receptors to lyse tumor cells [28]. The cell surface markers CD3, CD4, CD8, and CD19 are specific markers that distinguish T-cell, T-helper cells, cytotoxic T-cells, and B-cells, respectively [29,30].

Cancer vaccines should possess high affinity and stability of binding/cross reactivity with T-cell receptors (TCR), and efficiently recognize an endogenously-processed epitope expressed by tumor cells. We previously reported that SALF significantly inhibited HeLa cell growth in nude mice [10]. Based on this finding, we hypothesized that SALF combined with inactivated MBT-2 (murine bladder carcinoma cells) may hold potential as a cancer vaccine. Here, we examined its effect on the expression of immune-related genes and the recruitment of macrophages, lymphocytes, T-helper cells, and NK cells, before assessing the therapeutic and protective effects of as-designed adjuvant SALF on C3H/HeN mice bearing bladder cancer-derived tumors.

## 2. Results

### 2.1. SALF Inhibits Murine Bladder Tumor (MBT)-2 Cells

**Figure 1:**
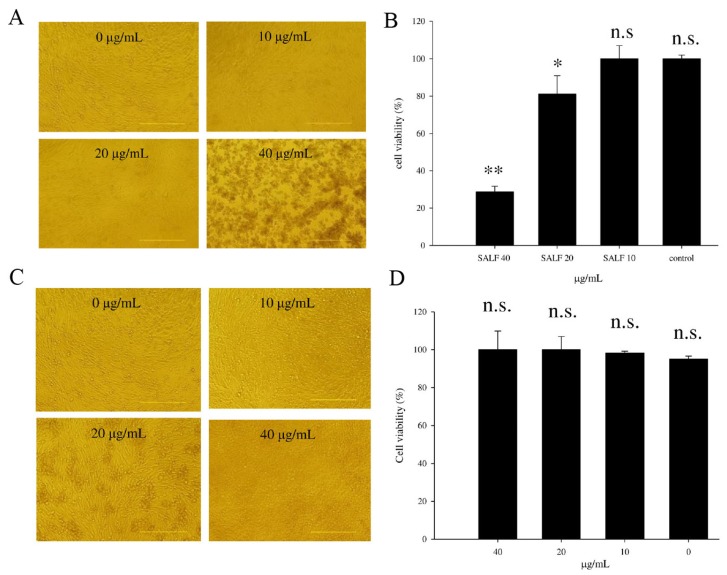
Cytotoxicity of MBT-2 lysate and shrimp anti-lipopolysaccharide factor (SALF) in mouse bladder tumor cells. (**A**,**B**) MBT-2 cells were treated with 0, 10, 20, or 40 μg/mL of SALF for 24 h; (**A**) Images showing cell morphology; (**B**) Cell viability measured by MTT assay; (**C**,**D**) MBT-2 cells were treated with 0, 10, 20 and 40 μg/mL of MBT-2 lysate for 24 h; (**C**) Images showing cell morphology; (**D**) Cell viability measured by MTT assay. Statistical comparisons were performed using Student’s *t*-test. *n.s.*: not significant; * *p* < 0.05; ** *p* < 0.01 *vs.* vehicle control.

In order to establish whether SALF is effective against bladder tumors, we examined the proliferation of MBT-2 cells treated with 0, 10, 20, or 40 μg/mL of SALF for 24 h (Figure 1A). Proliferation was unaffected by 10 μg/mL SALF, whereas cell proliferation was significantly inhibited at 20 and 40 μg/mL (Figure 1B). However, inactivated bladder carcinoma cells did not exhibit anticancer activities (Figure 1C,D).

### 2.2. Co-Treatment with SALF and Inactivated MBT-2 Lysate Promoted Innate IL-1β Production in Mouse Macrophages

**Figure 2:**
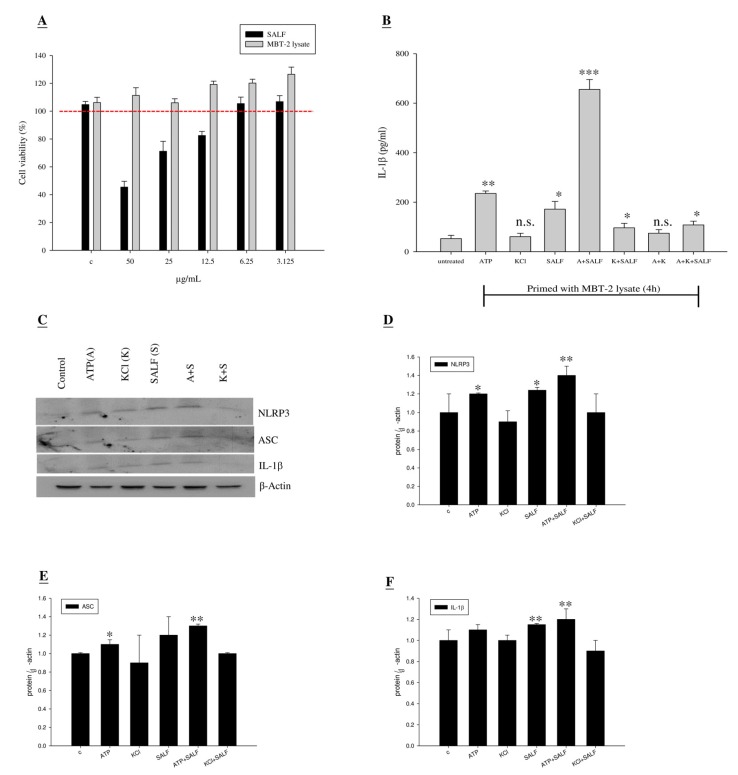
Co-treatment with SALF and inactivated MBT-2 lysate promoted innate IL-1β production in mouse macrophage (J774A.1) cells. (**A**) J774A.1 cells were treated with the indicated dose of SALF and MBT-2 lysate for 24 h, and cell viability was then measured by MTT assay; (**B**) Mouse macrophages (J774A.1) were plated onto 6-well plates at a density of 1 × 10^6^ cells per mL of RPMI-1640 media. Macrophages were primed by stimulation with 50 μg/mL per well of MBT-2 lysate for 4 h. As a positive control for NLRP3 inflammasome activation, 5 mM ATP was added to MBT-2 lysate-primed cells for 60 min. For inhibitor treatment, cells were pre-incubated for 60 min with 50 mM KCl before stimulation. Supernatant was removed at 24 h after SALF (6.25 μg/mL) stimulation, and IL-1b concentrations were quantified using ELISA. Six replicate wells were analyzed per assay. Results represent the mean ± SEM from three independent experiments. Statistical comparisons were performed using Student’s *t*-test. *n.s.*: not significant; * *p < 0.05*; ** *p < 0.01*; *** *p < 0.001 vs.* vehicle control; (**C**) Proteins isolated from cell lysates were subjected to Western blot with a specific antibody against the inflammasome; (**D**–**F**) Quantitative analyses of the blots shown in (**C**), with protein levels normalized to β-Actin. Results represent the mean ± SEM from three independent experiments performed in triplicate (Student’s *t*-test: *n.s.*: not significant; * *p < 0.05*; ** *p < 0.01 vs.* vehicle control).

Certain pathogen-derived immunomodulatory molecules can selectively promote adaptive immunity by activating antigen-presenting cells. Macrophages, APC cells capable of stimulating naive T-cell differentiation, are a key target of immunomodulatory molecules and adjuvants. Thus, we examined whether SALF can promote the Ag-specific Th1 response and facilitate production of IL-1β in J774A.1 (a murine macrophage line). To this end, we examined the level of IL-1β in J774A.1 cells treated with non-toxic SALF concentrations and inactivated MBT-2 lysate (Figure 2A). Levels of IL-1β were increased by treatment with 6.25 μg/mL SALF, when combined with inactivated MBT-2 lysate primed-stimulation (Figure 2B). Significantly higher levels of IL-1β were observed in macrophages as compared to untreated cells. In order to determine whether SALF controls inflammasome activation or affects the basal expression of inflammasome components and its effector proteins, expression levels of NLRP3, ASC, and IL-1β were analyzed by Western blot. The results suggest that SALF is involved in inflammasome activation, which can lead to NLRP3 and ASC activation, as well as IL-1β processing and secretion (Figure 2C–F).

### 2.3. SALF in Conjunction with Inactivated MBT-2 Lysate Modulates Tumor-Associated Chemokines and Cytokines

**Figure 3:**
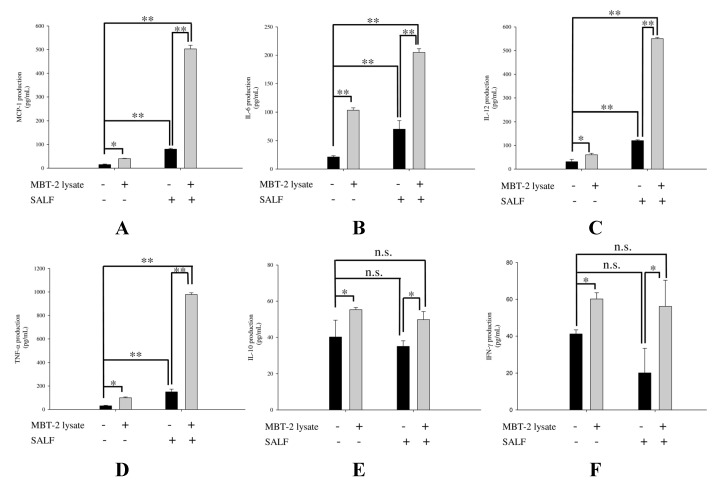
Co-treatment of J774A.1 cells with SALF and inactivated MBT-2 lysate induced release of MCP-1, IL-6, IL-12, and TNF-α. Monolayer J774A.1 cells were treated with or without 6.25 μg/mL of SALF for 24 h, following primed stimulation with 50 μg/mL MBT-2 lysate for 4 h; cytokine secretion was subsequently measured by ELISA. (**A**) MCP-1; (**B**) IL-6; (**C**) IL-12; (**D**) TNF-α; (**E**) IL-10; (**F**) IFN-γ. Values with different letters show significant differences (*p < 0.05*), as determined by ANOVA. The lowercase letters indicate the following: *n.s*., not significant; * significant (*p < 0.05*); ** significant (*p < 0.01*).

The chemokine MCP-1 enhances recruitment of monocytes and macrophages to tumor sites [17], while the proinflammatory cytokines IL-6, IL-10, and IL-12 have diverse effects on immunity; IL-6 influences T and B cell function [18], IL-12 stimulates the growth of activated natural killer (NK) cells, and CD8+ and CD4+ T-cells [19], and IL-10 modulates monocyte and macrophage function [20]. We proceeded to profile these chemokines and cytokines in J744A.1 cells treated with SALF for 24 h, in the presence or absence of inactivated MBT-2 lysate. We found that treatment with SALF (6.25 μg/mL) alone induced higher expression of cytokines (MCP-1, IL-6, IL-12, and TNF-α) compared with control, whereas levels of MCP-1, IL-6, TNF-α, and IL-12 were increased in cells co-treated with SALF and inactivated MBT-2 lysate (Figure 3). On the other hand, no significant differences in the expression of IL-10 and IFN-γ were observed.

### 2.4. Tumor Formation Is Attenuated by Immunization with SALF and Inactivated MBT-2 Lysate

**Figure 4:**
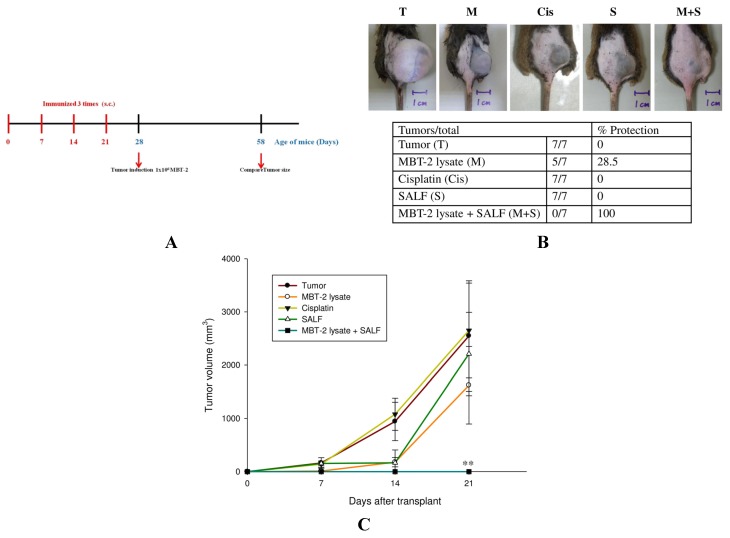
Immunization with SALF and inactivated MBT-2 lysate protects mice against tumorigenesis. (**A**) Schematic depicting the immunization timeline. Mice were immunized with PBS alone (Control), SALF alone (SALF), SALF with inactivated MBT-2 lysate (M + S), or inactivated MBT-2 lysate (M) at 7, 14, and 21 days; (**B**) Xenograft growth in mice (*n* = 13). After three immunizations, mice were injected with MBT-2 tumor cells (T) on the 28th day, and tumors were photographed on the 58th day; (**C**) Quantitation of tumor size at the indicated days after injection with MBT-2 tumor cells. Data are shown as mean ± SD. *n* = 13 mice/group and each value is an average of three independent experiments. * *p < 0.05*; ** *p < 0.01 vs.* vehicle tumor group.

Mice were immunized three times with SALF and/or inactivated MBT-2 lysate at 7, 14, and 21 days. The doses of SALF and inactivated MBT-2 lysate were determined through preliminary experiments (Supplementary Table S1). Tumor cells were then introduced by injecting MBT-2 cells into mice on the 28th day (a week after the third immunization) (Figure 4A). Co-treatment with SALF and the inactivated MBT-2 lysate significantly reduced tumor growth as compared to xenograft mice (i) immunized with inactivated MBT-2 lysate alone and (ii) mice treated with cisplatin (Figure 4B). In addition, tumor volume was diminished in mice immunized with SALF and inactivated MBT-2 lysate (Figure 4C).

### 2.5. Spleen Cell Activation is Modulated by Immunization with SALF and Inactivated MBT-2 Lysate

**Figure 5:**
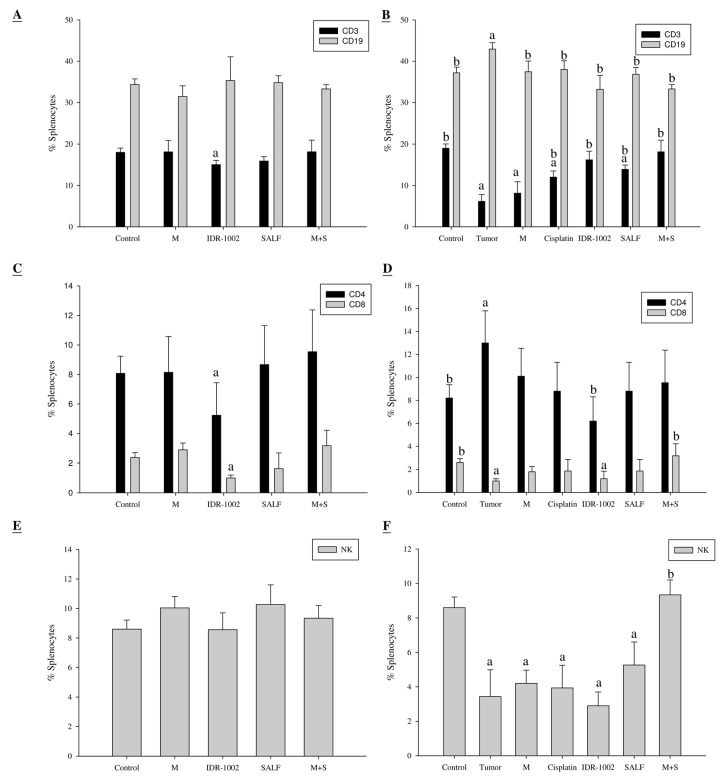
Immunization with SALF and inactivated MBT-2 lysate alters splenocyte subtypes. Mice were immunized three times with PBS (control; C), negative control (IDR-1002), inactivated MBT-2 lysate (M), SALF alone (SALF), SALF with inactivated MBT-2 lysate (M + S), or MBT-2 cells (tumor; T) at 7, 14, and 21 days of age. The immunized mice were challenged with MBT-2 cells on the 28th day, and splenocyte subtypes were analyzed using specific cell surface markers immediately after challenge (28 days, Figure 5**A**,**C**,**E**) and 30 days after challenge (58 days, Figure 5**B**,**D**,**F**). Data are shown as mean ± SD. *n* = 5 mice/group and each value is an average of three independent experiments. ^a^ *p < 0.05* *vs.* the control group; ^b^ *p < 0.05* *vs.* the tumor group.

Next, we examined the populations of B-cells, T-cells, helper T-cells, cytotoxic T-cells, and NK-cells in mice immunized with the vaccine (SALF + MBT-2 lysate). Mice treated with the vaccine did not exhibit any significant difference in CD3 and CD19 following the last immunization on day 28, as compared with the control (Figure 5A). We observed a significant increase in CD19 and decrease in CD3 in tumor-bearing mice, but no significant difference in the immunized group (M + S) after challenge on day 58, as compared to the control (Figure 5B). Mice treated with the vaccine exhibited no significant difference in CD4, CD8, or NK cells following the last immunization on day 28, as compared with the control (Figure 5C,E). The clinical use of cisplatin is often limited by its undesirable side effects, such as severe nephrotoxicity and hepatotoxicity. In this study, cisplatin treatment did not alter the splenocyte subtype percentage for CD19, CD4, or CD8 (Figure 5D), but decreased NK cell percentage at day 58, as compared to the control. Interestingly, the percentage of NK cells in mice immunized with inactivated MBT-2 lysate and SALF remained comparable to the control (Figure 5F). This phenomenon is also reflected in the tumor size.

### 2.6. Immunization with SALF and Inactivated MBT-2 Lysate Enhanced Infiltration of Immune Cells into the Tumor Site

**Figure 6:**
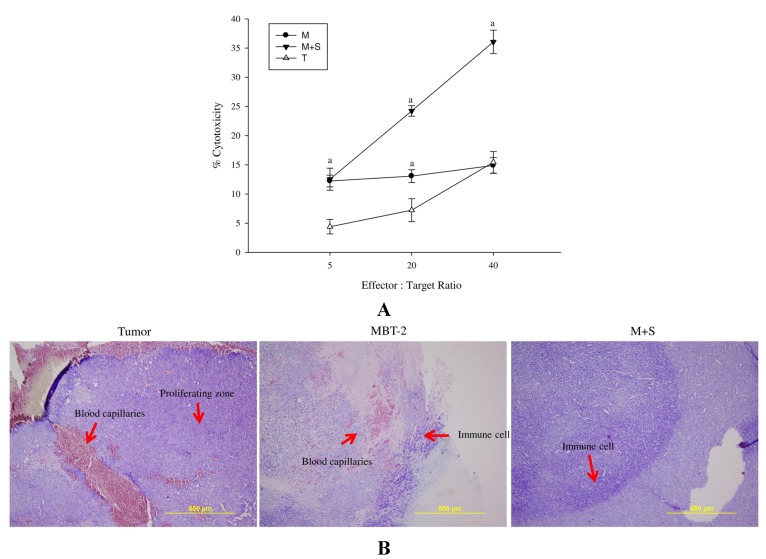
Immunization with SALF and inactivated MBT-2 lysate enhances cytotoxicity of T-lymphocytes and monocytes after challenge with MBT-2 on day 28, as analyzed on day 58. Mice were immunized three times with inactivated MBT-2 lysate (M), SALF with inactivated MBT-2 lysate (M+S), or MBT-2 cells (T) on 7, 14, and 21 days of age. The immunized mice were challenged with MBT-2 cells on the 28th day and analyzed on the 58th day. (**A**) Target cell death was determined by measuring the release of lactate dehydrogenase from lysed targets at various CTL:target ratios. Results represent the mean ± SEM from three independent experiments. Statistical comparisons were performed using Student’s *t*-test. *n.s.*: not significant; ^a^ *p < 0.05* *vs.* the tumor group; (**B**) Tumor tissues were subjected to Giemsa staining, and accumulation of lymphocytes and blood capillaries in the proliferating zone was observed. SALF prevented MBT-2-mediated induction of blood capillaries around the tumor proliferation region, and increased neutrophil accumulation.

Single-spleen-cell suspension effector cells were obtained from immunized and control mice, and used to analyze the cytotoxic ability of CTLs against MBT-2 cells. We observed that cells obtained from mice immunized with SALF and inactivated MBT-2 lysate had greater cytotoxicity than those from mice immunized with MBT-2 lysate alone (Figure 6A). Giemsa staining of the mice immunized with the vaccine also revealed enhanced infiltration of immune cells into the tumor site (Figure 6B), suggesting induction of tumor specific cytotoxicity and scavenging.

### 2.7. Immunization with SALF and Inactivated MBT-2 Lysate Activates Tumor-Associated Immune Cells

**Figure 7:**
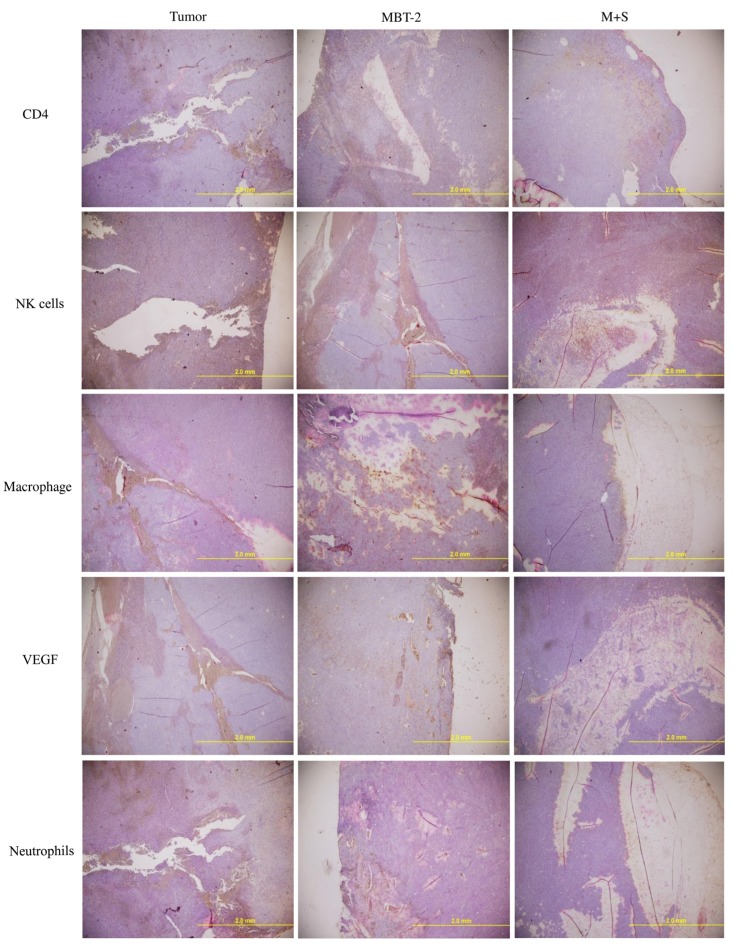
Immunization of MBT-2-challenged mice with SALF and inactivated MBT-2 lysate enhances T-helper cells, macrophages, and NK cells, and decreases VEGF and neutrophils. Mice were immunized as described in Figure 4. Cryosections of tumor sites were fixed in formaldehyde, and immunohistochemical analysis was performed using specific antibodies against T-cells, NK cells, macrophages, VEGF, and neutrophils.

In addition to cancerous cells, solid tumors are surrounded by extracellular matrix, non-malignant cells (e.g., fibroblasts and endothelial cells), and inflammatory cells (e.g., macrophages, neutrophils, mast cells, and lymphocytes) [21,22]. IHC analysis further indicated increased infiltration of T helper cells (CD4+), macrophages, and NK cells into the tumors of vaccinated mice, with a corresponding decrease of proliferation-associated VEGF (Figure 7). This suggests that vaccination regulated T-cell-associated antigens (TAA), as well as Tumor-associated macrophages (TAM).

## 3. Discussion

Antimicrobial peptides based on natural molecules exert antimicrobial effects through direct killing of pathogens or indirect modulation of the host defense system by enhancing immune responsive cells. Using immune molecules to elicit immune cells has been shown to be a promising means of vaccination for integrative cancer treatment [14]. The antimicrobial peptide shrimp anti-lipopolysaccharide factor (SALF) demonstrated herein is an effective immune molecule [10], complementing poor elicitation of tumor-associated cells [31]. Here, we report the suitability of SALF as a cancer vaccine component for use against murine bladder tumors.

Cancer vaccines should target antigens expressed preferentially by specific tumors. Cancer vaccination is constantly being developed and refined, in terms of antigen target identification and delivery, as well as immunomodulation [32]. Furthermore, the combination of multiple therapeutic approaches improves the efficiency of cancer treatment; the activation of immune cells or molecules through vaccination is a promising approach for integrative cancer treatment [14]. In addition to their prophylactic properties, cancer vaccines can act as therapeutic agents to combat existing cancer cells. In most cases, cancer vaccines are designed to be applied after the positive diagnosis of cancer; these vaccines should destroy malignant cells and prevent the reoccurrence of malignance [31].

In our report, the *in vitro* cytotoxicity of SALF was effective against bladder carcinoma cell line MBT-2. However, inactivated bladder carcinoma cells alone did not have anticancer activities (Figure 1). Although SALF-mediated immune responses and regulation of MAPK and NF-κB pathways have been well documented [33,34,35], such modulation of the levels of chemokines and cytokines by our vaccine (SALF in conjunction with inactivated MBT-2 lysate) for desired cancer immunity is unprecedented. SALF has been shown to possess adjuvant activity, whereby the NLRP3 inflammasome is formed in APC cells for production of inflammatory cytokine IL-1β, thereby promoting development of Th1 cells (Figure 2). Generally, treatment with the vaccine should increase the levels of MCP-1, IL-6, and IL-12, as shown in Figure 3. Of these effectors, MCP-1 likely concentrates monocytes and neutrophils at the tumor site, provoking an inflammatory response [17]; IL-6 and IL-12, which are responsible for activation of T- and B-cells, would facilitate production of tumor cell-specific antibodies [18,19]. Tumor-associated antigens (TAAs) are often recognized by host cytotoxic T lymphocytes (CTLs), and are known as CD8+ T-cells or killer T cell epitopes [14]. Host cytotoxic T lymphocytes (CTLs), also known as CD8t T-cells or killer T cells, recognize TAAs [14]. In the immunized mice, the absence of a significant increase in T-cell and CD-8 cells (Figure 5) signified an exceptional effect of the vaccination, as supported by the CTL assay (Figure 6A). The induction of CD8 T-cells supports the hypothesis that immunization with our vaccine activates an anti-tumor immune response. Furthermore, Giemsa staining revealed accumulation of immune cells at the MBT-2 cell injection site in immunized mice (Figure 6B). Efficient recognition of tumor cells by host effector cells in immunized mice may induce lymphocyte accumulation. Finally, immunohistochemical analysis of tumor sections revealed VEGF inhibition and neutrophil clearance in immunized mice, as well as a reduction of NK-cells, T-cells, and macrophages (Figure 7). In summary, the crustacean antimicrobial peptide SALF-based vaccine is concluded to be a highly suitable immune molecule for preventing murine bladder-associated tumors in mice.

## 4. Experimental Section

### 4.1. Cells and Mice

The murine carcinogen-induced bladder tumor cell line MBT-2 (derived from C3H mice) was cultured in Eagle’s minimum essential media supplemented with 10% heat inactivated fetal bovine sera [36]. The mouse macrophage J774A.1 cell line (ATCC, collection TIB-67) was cultured in Roswell park memorial institute media (RPMI-1640), as described previously [37]. MBT-2 cell-mediated tumors were induced in male C3H/HeN mice. All animal handing procedures were performed in accordance with National Taiwan Ocean University (NTOU) guidelines. All procedures were approved by the animal care and use committee of NTOU.

### 4.2. Peptides, Chemicals, and Reagents

The SALF (AC-ECKFTVKPYLKRFQVYYKGRMWCP-NH2) and IDR-1002 (VQRWLIVWRIRK-NH2) peptides were synthesized and purified by Genesis Biotech (Taipei, Taiwan) to a purity grade of >95%. Synthetic peptides were reconstituted in phosphate-buffered saline (PBS; pH 7.4) for the experiments. CD3 (Cat No. 553064, BD Biosciences, San Jose, CA, USA), CD19 (Cat No. 115505, BD Biosciences, San Jose, CA, USA), CD4 (Cat No. 100406, BioLegend, London, UK), CD8 (Cat No. 553064, BD Biosciences, San Jose, CA, USA), and NK cell marker (Cat No. 12-5971-82, eBioscience, San Jose, CA, USA) were purchased from the indicated companies. ELISA kits for MCP-1 (Cat No. 555260, BD Biosciences, San Jose, CA, USA), IL-1β (Cat No. 559603, BD Biosciences, San Jose, CA, USA), IL-6 (Cat No. 555240, BD Biosciences, San Jose, CA, USA), IL-10 (Cat No. 555252, BD Biosciences, San Jose, CA, USA), IL-12 (Cat No. 555256, BD Biosciences, San Jose, CA, USA), TNF (Cat No. 560478, BD Biosciences, San Jose, CA, USA) and IFN-γ (Cat No. 555138, BD Biosciences, San Jose, CA, USA) were used to determine cytokine levels. Antibodies against CD4 (NB110-97869, Novus Biologicals, Littleton, CO, USA), macrophages (Cat No. 550282, BD Biosciences, San Jose, CA, USA), NK cells (Cat No. 108901, BioLegend, London, UK), neutrophils (Cat No. 550291, BD Biosciences, San Jose, CA, USA), and VEGF (Cat No. 550549, BD Biosciences, San Jose, CA, USA) were used for immunohistochemistry (IHC).

### 4.3. Cell Proliferation

Cells were cultured at a density of 5 × 10^4^ cells per well in flat-bottomed 96-well plates, and supplemented with various combinations of AMPs. After three days, cell proliferation was assessed using the CellTiter 96^®^ AQueous One Solution Cell Proliferation Assay (Cat No. G3582, Promega, Madison, WI, USA), according to the manufacturer’s instructions. After four hours of incubation, cell viability was determined by measuring the absorbance at 490 nm.

### 4.4. Preparation of Tumor Vaccines and Immunization

MBT-2 cell lysates were inactivated by immersion in liquid nitrogen for 30 min, followed by immediate exposure to room temperature; this procedure was repeated three times. Immunization was performed using 100 μg of SALF in 100 μL phosphate buffered saline (PBS), with or without 50 μg of inactivated MBT-2 lysate. Vaccination was performed by subcutaneous injection at the ventral flank region. Primary immunization was performed on 7-day-old mice, and booster doses were injected at 14 and 21 days.

### 4.5. CTL Assay

The LIVE/DEAD^®^ Cell-Mediated Cytotoxicity Kit (L-7010, Invitrogen, New York, NY, USA) was used to analyze the toxicity of cytotoxic T-lymphocytes (CTLs). The CTL assay was performed on MBT-2 cells as previously described, with some modifications [38]. Briefly, cells were grown in RPMI 1640 (Gibco-BRL, Paisley, UK) supplemented with 10% fetal bovine serum (FBS; Dutscher, Brumath, France), 1 mM sodium pyruvate (Sigma, St. Louis, MO, USA), 100 UI/mL penicillin, 0.1 mg/mL streptomycin, and 1% glucose (complete medium). Target cells were cultured for 24 h before FACS analysis. For fluorescent labeling of target cells, 10 μL of 3 mM DIOC18 was mixed with 1 mL of complete media containing 10^6^ cells, and the mixture was incubated at 37 °C for 15 min, before being washed three times with PBS. Cell labeling and cell viability were confirmed by flow cytometry prior to the assay. For the cytotoxicity assay, single spleen cell suspensions were prepared from the spleen as effector cells. Red blood cells were lysed with ACK solution (150 mM NH_4_Cl, 1 mM KHCO_3_, 0.1 mM Na_2_EDTA (pH 7.4)). Effector cells were adjusted to 5 × 10^6^ cells/mL of complete media, and 100 μL of this dilution was mixed with 50 μL of target cell suspension (10^5^ cells/mL) to achieve E/T cell ratios of 5:1, 20:1, and 40:1. PI was then added (10 mg/mL). To enhance cell contact, the mixture was centrifuged, and the cell pellet was then incubated in complete media for 4 h at 37 °C in 5% CO_2_. Cell lysis was subsequently analyzed by flow cytometry (propidium iodide (10 mg/mL) was used to evaluate the viability of non-labeled and labeled target cells). Spontaneous cell death (*i.e.*, the 0% cell death baseline) was determined by incubating target and effector cells at 4 °C, in order to inhibit the cytotoxic activity of the effector cells. For maximum lysis (100% cell death), target and effector cells were incubated at 37 °C with 20 μL of saponin (0.3 mg/mL).

### 4.6. Tumor Growth Analysis

Mice were divided randomly into six groups (control, Tumor, MBT-2 lysate (M), Cisplatin (Cis), SALF (S) and MBT-2 lysate + SALF (M + S)), and each group consisted of thirteen mice. One week after the second booster vaccination, MBT-2 (1 × 10^6^ cells/mouse) was inoculated into the subcutaneous (*s.c.*) femoral left region of mice as described previously [39,40,41]. Cisplatin (Sigma-Aldrich, St. Louis, MO, USA) was dissolved in saline at a concentration of 1 mg/mL. Cisplatin was injected intraperitoneally at a dose of 4 mg/kg body weight (B.W.) on days 0, 7, and 14 (a total of three injections). Mice were sacrificed on day 28 or 58, and splenocyte subtypes were detected by flow cytometry. MBT-2 lysates and PBS alone were used as controls. Four weeks later, tumors were measured with calipers [42].

### 4.7. Immunohistochemistry

Tumor tissues were removed and fixed as previously described, with some modifications [31]. In brief, the sections were fixed with 4% formaldehyde. Tissue samples were stained with hematoxylin/eosin. Giemsa and IHC were analyzed by three independent investigators. Images were taken using a BX-51 microscope (Olympus, Tokyo, Japan).

### 4.8. SALF-Induced Cytokine Production in Vitro

Mouse macrophages (J774A.1) were plated in 6-well plates at a density of 1 × 10^6^ cells per mL of RPMI-1640 media. Macrophages were primed by culturing with 50 μg/mL MBT-2 lysate for 4 h at 37 °C with 5% CO_2_. For positive control wells, 5 mM ATP was added to MBT-2 lysate-primed cells for 60 min, and then SALF (6.25 μg/mL per well) was added for 24 h. For negative control wells, cells were incubated with 50 mM KCl for 60 min prior to stimulation. At the indicated time after stimulation, the supernatant was removed and IL-1β concentrations were quantified using ELISA. Proteins isolated from cell lysates were probed with a specific Ab against the inflammasome for Western blot.

### 4.9. Western Blot Analysis

IL-1β, ASC, caspase-1, and NLRP3 protein were detected in the cell lysates of stimulated mouse macrophages. Whole protein was extracted using the methanol/chloroform method. Samples were then boiled (95–99 °C) for 5 min, and loaded onto an SDS-PAGE gel (15%) with Protein ladder (10–170 kDa, Precision Plus Protein Standards). Proteins were transferred to nitrocellulose membranes using a semi-dry Western transfer system. Membranes were subsequently blocked using 1% BSA in TBS for 1 h, washed, and then incubated at 4 °C overnight with antibodies against IL-1β (Cat No. sc-7884, Santa Cruz Biotechnology, TX, USA), ASC (Cat No. sc-22514-R, Santa Cruz Biotechnology, Dallas, TX, USA), and NLRP3 (Cat No. sc-34411, Santa Cruz Biotechnology, Dallas, TX, USA). Membranes were washed and incubated with the specified secondary antibody (Santa Cruz Biotechnology) for 1 h. Blots were developed using chemiluminescence reagent (Cat No. 79907, Abcam, Cambridge, MA, USA).

### 4.10. Statistical Analysis

All experiments were performed in triplicate on three biological replicates. All data are presented as mean ± SEM. Two-tailed *t* tests were performed to identify significant differences between two groups. Analyses of multiple groups were performed by one-way ANOVA with Bonferroni post tests, using GraphPad Prism Version 5. For all statistical tests, *p* values of less than 0.05 were considered to be statistically significant. Histological and *in vivo* data are representative of three independent experiments. Each vaccination treatment condition used a group of 13 mice, and the experiments were repeated three times.

## 5. Conclusions

Shrimp anti-lipopolysaccharide factor (SALF), an antimicrobial peptide, has been reported to possess anticancer activity. The observation that T-cell antigens recognize tumor cells opened the door to immunotherapy in the modality of cancer prevention. Peptide molecules boosting anti-tumor cell immunity may serve as immuno-therapeutic agents and suppress tumor progression. Here, a cancer vaccine comprised of SALF and inactivated murine bladder carcinoma cell (MBT-2) lysate was tested in mouse tumor models. The presence of SALF in the cell culture media inhibited the progression of MBT-2 *in vitro*. Moreover, SALF has also been shown to possess adjuvant activity; it promotes NLRP3 inflammasome formation in APC cells for the production of the inflammatory cytokines IL-1β, MCP-1, IL-6, and IL-12 in mouse macrophages, thereby selectively promoting adaptive immunity by activating antigen-presenting cells. Immunization with SALF and inactivated MBT-2 lysate prevented the formation of MBT-2 cell-mediated tumors, and enhanced T-cell, cytotoxic T-cell, and NK cell activation. Furthermore, recruitment of monocytes, lymphocytes, T-helper cells, and NK cells was increased, while expression of VEGF was decreased in immunized mice. Thus, our SALF-based vaccine counters murine bladder-associated tumors in mice, and is therefore a potential candidate for future studies into novel cancer treatments.

## Supplementary Files

Supplementary File 1
