# Supplementary material for: Enhanced Control of Bladder-Associated Tumors Using Shrimp Anti-Lipopolysaccharide Factor (SALF) Antimicrobial Peptide as a Cancer Vaccine Adjuvant in Mice
Source: Mar Drugs. 2015 May 21;13(5):3241–58. doi: 10.3390/md13053241 (PMC4446627; doi:10.3390/md13053241)
Supplement: Supplementary File 1 [file marinedrugs-13-03241-s001.pdf]

Supplementary Information

Table S1. Mice received three doses of vaccine, and were injected with MBT-2 tumor cells on the 28th day. Tumor volume was measured using calipers on the 7th, 14th, and 21st day (Group of 5 mice; *n* = 3).

Group	0 (Day)	7 (Day)	14 (Day)	21 (Day)
**M50**	0	10.40 ± 7.12	177.80 ± 18.23	1622.80 ± 126.70
**M100**	0	20.70 ± 6.23	255.20 ± 20.21	1802.10 ± 132.20
**M200**	0	13.70 ± 7.21	323.20 ± 55.30	1900.30 ± 178.20
**M200 + S200**	0	9.30 ± 7.12	157.80 ± 45.20	322.80 ± 27.30
**M200 + S100**	0	20.40 ± 13.5	144.20 ± 55.10	223.60 ± 23.40
**M200 + S50**	0	32.10 ± 3.10	110.40 ± 26.10	244.20 ± 38.20
**M100 + S200**	0	5.40 ± 4.22	90.20 ± 32.20	260.10 ± 34.20
**M100 + S100**	0	11.20 ± 1.70	101.20 ± 45.50	230.20 ± 20.10
**M100 + S50**	0	23.10 ± 2.95	84.20 ± 32.50	250.20 ± 42.50
**M50 + S200**	0	7.40 ± 4.32	41.80 ± 11.20	170.80 ± 34.20
**M50 + S100**	0	0.80 ± 0.10	20.60 ± 9.20	115.00 ± 32.50
**M50 + S50**	0	5.60 ± 2.54	50.20 ± 17.20	180.10 ± 23.50
**T**	0	163.00 ± 99.00	944.20 ± 361.20	2858.40 ± 695.60

© 2015 by the authors; licensee MDPI, Basel, Switzerland. This article is an open access article distributed under the terms and conditions of the Creative Commons Attribution license (http://creativecommons.org/licenses/by/4.0/).
